# Do polygenic risk and stressful life events predict pharmacological treatment response in obsessive compulsive disorder? A gene–environment interaction approach

**DOI:** 10.1038/s41398-019-0410-0

**Published:** 2019-02-04

**Authors:** María Alemany-Navarro, Javier Costas, Eva Real, Cinto Segalàs, Sara Bertolín, Laura Domènech, Raquel Rabionet, Ángel Carracedo, Jose M. Menchón, Pino Alonso

**Affiliations:** 1 Institut d’ Investigació Biomèdica de Bellvitge (IDIBELL), L’Hospitalet de Llobregat (Barcelona), Spain; 2 OCD Clinical and Research Unit, Psychiatry Department, Hospital Universitari de Bellvitge, L’Hospitalet de Llobregat (Barcelona), Spain; 3 Grupo de Xenética Psiquiátrica, Instituto de Investigación Sanitaria de Santiago, Complexo Hospitalario Universitario de Santiago de Compostela, Servizo Galego de Saúde, Santiago de Compostela, Spain; 4 Centre for Genomic Regulation (CRG), The Barcelona Institute of Science and Technology, Dr. Aiguader 88, Barcelona, 08003 Spain; 5 Universitat Pompeu Fabra (UPF), Barcelona, Spain; 6 CIBER in Epidemiology and Public Health (CIBERESP), Barcelona, Spain; 7 Grupo de Medicina Xenómica, Universidade de Santiago de Compostela, Centro Nacional de Genotipado - Instituto Carlos III, Santiago de Compostela, Spain; 8 Centro de Investigación Biomédica en Red de Enfermedades Raras, Santiago de Compostela, Spain; 9 CIBERSAM (Centro de Investigación en Red de Salud Mental), Instituto de Salud Carlos III, Madrid, Spain; 10 Department of Clinical Sciences, Bellvitge Campus, University of Barcelona, Barcelona, Spain

## Abstract

The rate of response to pharmacological treatment in Obsessive-compulsive disorder (OCD) oscillates between 40 and 70%. Genetic and environmental factors have been associated with treatment response in OCD. This study analyzes the predictive ability of a polygenic risk score (PRS) built from OCD-risk variants, for treatment response in OCD, and the modulation role of stressful life events (SLEs) at the onset of the disorder. PRSs were calculated for a sample of 103 patients. Yale–Brown Obsessive Compulsive Scale (YBOCS) scores were obtained before and after a 12-week treatment. Regression analyses were performed to analyze the influence of the PRS and SLEs at onset on treatment response. PRS did not predict treatment response. The best predictive model for post-treatment YBOCS (post YBOCS) included basal YBOCS and age. PRS appeared as a predictor for basal and post YBOCS. SLEs at onset were not a predictor for treatment response when included in the regression model. No evidence for PRS predictive ability for treatment response was found. The best predictor for treatment response was age, agreeing with previous literature specific for SRI treatment. Suggestions are made on the possible role of neuroplasticity as a mediator on this association. PRS significantly predicted OCD severity independent on pharmacological treatment. SLE at onset modulation role was not evidenced. Further research is needed to elucidate the genetic and environmental bases of treatment response in OCD.

## Background

Obsessive-Compulsive Disorder (OCD) is a complex neuropsychiatric condition that may interfere severely in the patient’s life and lead to maladaptive behavior^1,2^. Lifetime prevalence of the disorder is 2–3%^3^. Treatment usually consists of medication and cognitive-behavioral therapy, as recommended in OCD clinical guidelines^4^.

Selective serotonin reuptake inhibitors (SSRIs) comprise the first-line pharmacological treatment for OCD. However, treatment response is variable, with the proportion of OCD patients that improve with pharmacological treatment oscillating between 40 and 70%, and in some cases only a partial response is obtained^5^. Non-responders may be treated with another SSRI^6^, with antipsychotics as adjunctive treatment^7^, or clomipramine either as monotherapy or as a coadjuvant^8^.

Several studies have examined the genetic and environmental factors associated with treatment response in OCD. Genetic variants in catecholaminergic (*HTR2A, HTR1B, SLC6A4, COMT*), glutamatergic (*SLC1A1, GRIN2B, DLGAP2, SLITRK5, DLGAP1*), and neurotrophic (*BDNF*) systems have been associated with response to SSRIs^9–13^. Some, but not all, of these genes have been related to increased risk of developing OCD (*BDNF*^14–16^, *SLC1A1*^17^, *COMT*^18^, *SLC6A4*^19^). Meanwhile, pharmacokinetic studies have also associated variants of an enzyme from the cytochrome P450 family (*CYP2D6*) with response to SSRIs and non-selective serotonin reuptake inhibitors (SRIs)^20,21^. Besides candidate gene association studies, a genome-wide association study (GWAS) has been carried out to study SSRI response in OCD. Although only one variant (located near the DISP1 gene) met the genome-wide significance level, other 41 presented indications of association; most of these variants played a role in the glutamatergic and serotonergic pathways^22^. These results suggest that genetic factors may influence treatment response in OCD.

GWAS data can also be used to generate an important index known as the polygenic risk score (PRS), a measure of an individual’s genetic risk conferred by common variants of the studied trait. Based on the results of a GWAS discovery sample, it is calculated as the sum of the number of risk alleles carried by an individual, weighted according to their effect^23,24^.

The association between a PRS and treatment response has been analyzed in several psychiatric conditions, including affective disorders and schizophrenia^25^. In the case of SSRI response, inconsistent data have been reported for affective disorders. As an example, researchers involved in the GENDEP, STAR*D, and MARS projects constructed a PRS for antidepressant response derived from a meta-analysis of GENDEP and MARS participants. This PRS accounted for up to 1.2% of response variance in the STAR*D sample^26^. Conversely, García González et al^27^. constructed a PRS for treatment response in major depressive disorder (MDD) using pharmacogenetic samples from the GENDEP and STAR*D studies, but found no significant associations. To the best of our knowledge, PRS methodology has not been employed to study treatment response in OCD.

Stressful conditions have also been observed to influence treatment response in several mental disorders. One of the most extensively studied of these is childhood abuse, which has been associated with treatment response in depression^28–31^ and social anxiety disorder^32^. A history of trauma has also been related to greater treatment resistance in OCD^33^. Conversely, Shavitt et al^34^. found that OCD patients reporting trauma benefited more from SSRI treatment than those who did not. Further research has investigated the influence of gene–environment interaction (GxE) on treatment response. Thus, it has been reported that response to SSRIs in affective disorders is influenced by the interaction between non-traumatic but stressful life events (SLEs) and variants of the *SLC6A4*^35^, *BDNF* and *ST8SIA* (a gene involved in neuroproliferation and neuroplasticity) genes^36^. In addition, a gene associated with the catecholamine system (*SLC6A2*) has been related to treatment response in MDD, through an interaction with childhood abuse^37^. Finally, a GxE influence on treatment response has also been described in OCD for variants of the glutamatergic gene *SLC1A1* and SLEs at onset of the disorder^12^. However, none of these studies has made use of a PRS.

Given the above, the objective of this study was to analyze how genes influence treatment response in OCD, and how this influence may be modulated by certain environmental conditions. We hypothesized that a PRS built for OCD would predict treatment response to SRIs and that this association would be modulated by SLEs at onset of the disorder.

## Methods

### Subjects

Our study sample comprised 103 Spanish Caucasian patients (46 females; mean age 33.23 ± 9.66) recruited from the OCD clinic at Bellvitge Hospital (Barcelona, Spain). All participants met the DSM-IV criteria for OCD diagnosis^38^, with a duration of at least one year. Diagnoses were independently assigned by two psychiatrists with extensive clinical experience in OCD, each of whom interviewed the patients separately using the Structured Clinical Interview for DSM-IV Axis I Disorders-Clinician Version (SCIDCV)^39^. Patients were offered treatment at our clinic with a SRI (SSRI or clomipramine), considering their history of previous medication and preference. Maximum recommended doses were administered to the patients for a minimum period of 12 weeks. Exclusion criteria consisted of presenting psychoactive substance abuse/dependence (either current or in the past six months), psychotic disorders, intellectual disability, severe organic or neurological pathology except tic disorder, and autism spectrum disorder. Other affective and anxiety disorders were not considered criteria for exclusion if OCD was the main diagnosis.

After receiving a full description of the study, patients were required to give their written informed consent. This study was performed in accordance with the Helsinki Declaration of the World Medical Association, and approved by the Ethical Committees of Bellvitge Hospital.

### Clinical assessment

Participants’ sociodemographic and clinical characteristics and medical data were collected in structured interviews during their first visit to our unit. Age at onset was defined as the moment when obsessive symptoms reached a clinically significant level. Baseline severity of the obsessive and compulsive symptoms was assessed through the clinician-administered version of the Yale-Brown Obsessive Compulsive Scale (Y-BOCS)^40^. The presence of obsessive/compulsive dimensions in the worst-ever period of the disorder was evaluated using the DY-BOCS scale^41^. Family psychiatric history was coded in percentages for each category, considering solely family members who had received a formal diagnosis.

#### Treatment response

Treatment response was assessed by means of a clinician-administered version of the Y-BOCS^40^. Patients were assessed twice: at baseline and after 12 weeks at maximum tolerated SRI doses, in both cases analyzing the severity of obsessive and compulsive symptoms.

#### Stressful life event assessment

Life stress at onset of the disorder was analyzed using the methodology described in Real et al.^12^. In face-to-face interviews, patients were asked whether they could identify a SLE in the year prior to the onset of the disorder. Patients reporting a SLE at onset were assigned to the SLE-preceded OCD group, while the rest of the patients formed the non-SLE-preceded OCD group. Patient-reported SLEs at onset were contrasted with the Paykel Scale of Stressful Life Events^42^ to confirm that these coincided with any of the 61 events listed on the scale.

#### Current treatment

Thirty-six (35.0%) of the 103 patients received SSRIs (sertraline, fluoxetine, fluvoxamine, paroxetine, citalopram or escitalopram) as monotherapy according to their previous history of response. The remaining 67 (65.0%) were administered clomipramine as monotherapy due to a previous history of resistance to at least three SSRIs.

### Genotyping data

All genotyping, quality control, and imputation procedures were carried out on an original sample consisting of 433 OCD patients and 484 controls, and are described elsewhere^43^. Genotypic data for cases and controls were obtained using the Axiom Exome array (Affymetrix, Santa Clara, CA, USA), following the manufacturer’s instructions. This panel includes 300 000 variants mainly from coding regions (85%), a type of variation that contributes disproportionately to single nucleotide polymorphism (SNP) heritability^44–46^. Variant call was performed using Affymetrix genotyping console software with the Axiom GT1 algorithm.

Quality control procedures were carried out using PLINK. SNPs that did not pass the following filters were removed from the study: (i) genotyping call rate greater than 95%; (ii) non-significant departures from Hardy-Weinberg equilibrium in control sample (*P* > 1 × 10^−3^); (iii) non-significant difference in call rate between cases and controls (P > 1 × 10^−3^).

### Composition of the polygenic risk score

In order to generate a discovery sample, 131 samples that would constitute the target sample to study response to treatment, were removed from the genotyping data set. The target sample was selected considering the availability of neuroimaging data from these patients for another study currently underway. Then, an allelic association test for OCD status was carried out on the discovery sample using PLINK v1.07^47^, considering all the autosomal SNPs at MAF > 5%. These SNPs were all included in the PRS model. The PRS in the target sample was calculated as the sum of every risk allele carried by an individual, weighted according to its effect size, measured as the logOR (using the score option in PLINK v1.07). Several different P _threshold_, from 0.01 to 1 (that is, inclusion of all the SNPs) were considered.

### Statistical analyses

Variance analyses were carried out using generalized linear models (GLM) to analyze the predictive power of the PRS for treatment response, and subsequently, the modulatory role of SLEs at onset in this relation. Basal and post-treatment Y-BOCS scores (basal Y-BOCS and post Y-BOCS) were included in the equation separately. Basal Y-BOCS was considered an independent variable, and post Y-BOCS, the dependent variable. An analysis of post Y-BOCS distribution was performed in order to select a link function for it in the GLM. The model was adjusted for sex, age, history of pharmacological resistance, age at onset, and family psychiatric history of OCD, given the observed influence of these factors on OCD phenotype and treatment response^48–50^. The Akaike information criterion (AIC) was compared between the different models executed in order to determine which showed the best fit to the data. Given the large differences between the variables’ scales, all of them were standardized in order to obtain interpretable results.

To rule out any possible correlation between the PRS and the adjustment variables, variance analyses were carried out (t-test/ANOVA or Pearson correlation according to the qualitative or quantitative nature of the variables, respectively). Different models were used to analyze the predictive power of PRS for treatment response (Table 1). First, only basal Y-BOCS was included as an independent variable in order to analyze a possible association between both Y-BOCS measures (*Model 1*, Table 1). Then, a new analysis (*Model 2*, Table 1) was performed that included the PRS as an independent variable together with basal Y-BOCS. *Model 2* was then readjusted for each of the eight abovementioned demographic and clinical variables (*Model 3*, Table 1). Furthermore, since the PRS was built from OCD-risk variants, we thought it might also predict disorder severity^51^ (in addition to disease status: ^52,43^). Then, further models were executed to analyze a possible association between each of the Y-BOCS measures and the PRS (*Models 4* and *5*, Table 1). When basal Y-BOCS was a dependent variable (*Model 5*), its distribution was analyzed in order to select an appropriate link function in the GLM.

**Table 1:** Models analyzing predictors for treatment response

*Model 1*	Post Y-BOCS ~ Basal Y-BOCS
*Model 2*	Post Y-BOCS ~ Basal Y-BOCS + PRS
*Model 3*	Post Y-BOCS ~ Basal Y-BOCS + PRS + Adjustments
*Model 4*	Post Y-BOCS ~ PRS
*Model 5*	Basal Y-BOCS ~ PRS
*Model 6*	Post Y-BOCS ~ Basal Y-BOCS + PRS + SLE
*Model 7*	Post Y-BOCS ~ Basal Y-BOCS + PRS + SLE + Adjustments

*Y-BOCS* Yale–Brown obsessive-compulsive scale, *PRS* polygenic risk score, *SLE* stressful life event

With respect to SLEs at onset, the possibility of significant differences in PRS between those presenting a SLE at onset or not was ruled out by means of a t-test. Correlations between SLEs at onset and each demographic or clinical variable included in the models as independent variables (including basal Y-BOCS), were also tested using t-test analysis for continuous variables and chi square tests, for categorical variables.

To explore the modulatory role of SLEs at onset in treatment response, a new model was constructed that included SLEs at onset as an independent variable together with basal Y-BOCS and the PRS (*Model 6*, Table 1). All analyses were performed using R software version 3.2.2 (R Foundation for Statistical Computing, Vienna, Austria).

## Results

### Genotyping and quality control

Quality control filtering yielded genotypic data from a sample of 370 cases and 443 controls. The final data set consisted of a total of 38 305 SNPs at a frequency > 5% in the total sample. Further details on application of the quality control filters are given in Costas et al.^43^.

### Comparison with previous GWAS

Our results were compared with the available data from previous GWAS studies: SNPs at *P* < 01 × 10^−3^ in meta-analysis of Stewart et al.^53^. and SNPs at *P* < 01 × 10^−4^ in the study of Matthiesen et al.^54^. None of the 33 SNPs reported by Matthiesen et al. were presented in our samples. Forty-four SNPs present in our samples are among the 601 reported by Stewart et al. Two of them are at *P* < 0.05 in our samples, but only one, rs6845865, showed the same direction of association.

### Polygenic risk score

A PRS was constructed for a total of 37 523 autosomal SNPs associated to OCD status in the discovery sample. One-hundred and three from the original 131 patients conformed the final target sample.

### Treatment response

Three patients out of the 103 that constituted the target sample did not complete the 12-week treatment. Then, treatment response was obtained for a final sample of 100 patients (44 females; mean age 33.42 ± 9.67) (Table 2). The Patients’ scores on the Y-BOCS scale before and after the treatment period are shown in Table 3.

**Table 2:** Sodiodemographic and clinical characteristics of the sample of 100 OCD patients

Age, Years, Mean ± SD (Range)	33.42 ± 9.67 (18–57)			
Male/Female, *n* (%)	56/44 (56.0%, 44.0%)			
Age at Onset of OCD, Mean ± SD (Range)	21.62 ± 10.03 (6–70)			
*Baseline Y-BOCS Score, Mean* *±* *SD (Range)*				
Global	26.26 ± 5.16 (11–36)			
Obsessions	13.12 ± 3.24 (0-26)			
Compulsions	12.98 ± 2.99 (0- 19)			
*Baseline HDRS, Score, Mean* *±* *SD (Range)*	12.7 ± 5.05 (2–28)			
*Current Comorbid Diagnosis, n (%)*				
No comorbid disorder	53 (53.0)			
Other Anxiety Disorder	10 (10.0)			
Mood Disorder	9 (9.0)			
Tics	3 (3.0)			
Eating Disorders	2 (2.0)			
Other	23 (23.0)			
*Presence of Dimensions in worst-ever period n (%)*				
Aggresive/checking	70 (70.0)			
Symmetry/Ordering	43 (43.0)			
Contamination/cleaning	49 (49.0)			
Hoarding	26 (26.0)			
Miscelaneos	32 (32.0)			
*History of Pharmacological Resistance n (%)*				
1 SSRI	27 (27.0)			
2 SSRIs	12 (12.0)			
>2 SSRIs	61 (61.0)			
*Family Psychiatric History n (% of patients)*	1st grade	2nd grade	3rd grade	Total
No psychiatric diagnosis	47 (47.0)	38 (38.0)	80 (80.0)	30 (30.0)
OCD	2 (2.0)	11 (1.0)	1 (1.0)	10 (10.0)
Subclinic OCD	6 (6.0)	5 (5.0)	1 (1.0)	12 (12.0)
Anxiety disorder different from OCD	13 (13.0)	4 (4.0)	0 (0.0)	15 (15.0)
Mood disorder	14 (14.0)	28 (28.0)	1 (1.0)	28 (28.0)
Psychotic disorder	5 (5.0)	5 (5.0)	8 (8.0)	13 (13.0)
Drug abuse	8 (8.0)	2 (2.0)	3 (3.0)	10 (10.0)
Eating disorder	1 (1.0)	0 (0.0)	0 (0.0)	1 (1.0)
Tics/ Guilles de la Tourette	2 (2.0)	5 (5.0)	4 (4.0)	9 (9.0)
Others	2 (2.0)	2 (2.0)	2 (2.0)	5 (5.0)

*OCD* obsessive-compulsive disorder, *Y-BOCS* Yale–Brown obsessive-compulsive scale, *HDRS*, Hamilton depression rating scale

**Table 3:** Y-BOCS scores before and after 12-week treatment period

*Y-BOCS Score, Mean* *±* *SD (Range)*		
	*Basal YBOCS*	*Post YBOCS*
*Global*	26.26 ± 5.16 (11–36)	19.18 ± 6.80 (6–36)
*Obsessions*	13.12 ± 3.24 (0–26)	9.49 ± 3.46 (3–18)
*Compulsions*	12.98 ± 2.99 (0–19)	9.57 3.60 (0–18)

*Y-BOCS* Yale–Brown obsessive-compulsive scale

Regression analyses were carried out in a final sample of 100 patients, for which treatment response was obtained.

#### Polygenic risk score prediction ability

The main results are summarized in Table 4. Variance analysis for the PRS and adjustment variables did not reveal any significant results. Basal Y-BOCS was a significant predictor of post Y-BOCS (*Models 1* and *2*; Table 4, Supplemental Figure S1), even when including the adjustment variables. Among these latter, only age was a significant predictor of post-treatment Y-BOCS (*Model 3*, Table 4). However, age did not maintain its predictive power when the rest of the adjustment variables were included in the model (*P* = .11). The PRS was not a predictor of post Y-BOCS in any of these models.

**Table 4:** Predictors for treatment response

	*Model 1*. Post Y-BOCS regressed on _Basal Y-BOCS_			*Model 2*. Post Y-BOCS regressed on _Basal Y-BOCS and PRS_			*Model 3*. Post Y-BOCS regressed on _Basal Y-BOCS, PRS and Age._		
	_*B*_	_CI_	_*p*_	_*B*_	_CI_	_*p*_	_*B*_	_CI_	_*p*_
*(Intercept)*	−2.04E-16	−0.13 to 0.13	1.000	0.01	−0.13 to 0.13	1.000	−3.83E-17	−0.13 to 0.13	1.000
*Basal Y- BOCS*	0.74	0.61 to 0.87	<.001*	0.73	0.60 to 0.87	<.001*	0.75	0.62 to 0.89	<.001*
*PRS*				0.04	−0.10 to 0.18	.552	0.03	−0.11 to 0.16	.691
Age							0.17	0.04 to 0.30	.009*
Observations		100			100			100	
*R*^2^/adj. *R*^2^		.551/.547			.553/.544			.581/.568	
AIC		208.677			210.324			205.691	

The predictive abilities of Basal YBOCS, PRS and Age for Post YBOCS arepresented as Beta coefficients (β) from the regressions as well as with theproportion of variance explained by the models (*R*^2^/Adj. *R*^2^). The model fit isalso reported by the Akaike Information Criterion (AIC). *Y-BOCS* Yale-Brown obsessive-compulsive scale, *PRS* polygenic risk score. *p < 0.05

Although the models barely differed from one another, *Model 3* showed the best fit (AIC = 205.691) and explained the highest proportion of variance (*R*^2^ = .581; adj. *R*^2^ = .568) (Table 4). Considering that Model 3 seemed the best model and the PRS was not a predictor of post Y-BOCS, a new model was tested removing the PRS and maintaining age as an independent variable (Supplemental Table S1). The AIC for this new model was lower than that for *Model 3* (Table 4), presenting a better fit and explaining a relatively similar percentage of the variance (*R*^2^ = .575; adj. *R*^2^ = .566).

In light of the above, the PRS was regressed on both Y-BOCS measures separately. The PRS seemed to significantly predict both post and basal Y-BOCS (Table 5). The AIC for *Model 4* (AIC = 283.494) was higher than that for all previous models, thus showing a worse fit and explaining quite a low percentage of variance compared with the previous models (*R*^2^ = .052; adj. *R*^2^ = .043) (Table 5).

**Table 5:** PRS predictive ability for YBOCS scores

	*Model 4*. Post-YBOCS regressed on PRS			*Model 5*. Basal Y-BOCS regressed on PRS		
	*β*	CI	*p*	*β*	CI	*p*
(Intercept)	−4.37E-16	−0.19 to 0.19	1.000	−3.18E-16	−0.19 to 0.19	1.000
PRS	0.23	0.04 to 0.42	.020*	0.25	0.06 to 0.44	.009*
Observations	100	100				
*R*^2^ / adj. *R*^2^	.052 /.042	.064 /.055				
AIC	283.494	282.118				

The predictive abilities of PRS for Post and Basal Y-BOCS are displayed as Beta values (β) from the regressions and the proportions of variance explained by the models (*R*^2^/Adj. *R*^2)^. The model fit is also reported by the Akaike Information Criterion (AIC)

*Y-BOCS* Yale–Brown obsessive-compulsive scale, *PRS* polygenic risk score

*p < .05

#### SLE at onset modulation ability

Of the final target sample of 100 patients, 40 (40%) were categorized as SLE-preceded OCD and 60 (60%) as non-SLE-preceded OCD.

ANOVA analyses of the PRS and SLEs at onset revealed significant differences in the PRS between those reporting a SLE at onset of the disorder and those who did not (*t* = 2.96; d*f* = 98; *P* = .004). In addition, significant associations were found between a SLE at onset and age at onset (*t* = 2.29; d*f* = 98; *P* = .024) and sex (*χ*^*2*^ = 4.93; *df* = 1; *P* = .039). These results are shown in Supplemental Tables S2 and S3.

According to the modulation analysis, a SLE at onset was not a predictor for post Y-BOCS when considering basal Y-BOCS and the PRS (*B* = -0.02; 95% CI, -0.29 to 0.26; *P* = .911), nor when any of the adjustment variables were included.

## Discussion

This study was conducted to analyze if a PRS composed of OCD-risk genetic variants predicted treatment response in a sample of OCD patients, and whether this relation was modulated by SLEs at onset. According to the main regression analysis, the PRS did not predict treatment response, and basal Y-BOCS was the best predictor of post Y-BOCS in all cases. Only age, out of all the adjustment variables, showed a predictive capacity for treatment response, as those models that included it presented the best fit. An analysis of OCD severity indicated that the PRS was a predictor of both basal and post Y-BOCS. The results of a modulation analysis suggested that SLEs at onset did not play a significant role in treatment response when considering the PRS. Differences in SLE at onset prevalence were observed for the PRS, sex, and age at onset, whereby patients with a higher genetic risk, women, and those presenting a later age at onset were more likely to report a SLE at onset.

With respect to the regression analysis, PRS was not a predictor for treatment response in our sample. Some evidences exist about the non-shared genetic bases of OCD risk and OCD treatment response^22,55^. In addition, although other studies have found an association between OCD treatment response and genes previously associated to OCD risk^14,15,19^ (such as *SLC1A1, SLC6A4* or *BDNF*^12,13^), to our knowledge, only one genetic variant (rs3087879 in SLC1A1 gene) has been related to both OCD treatment response (a greater resistance) and OCD risk^12^. We do not know to what extent this is due to different genetic bases of treatment response and OCD risk, since the variants studied in candidate gene association studies for OCD risk do not usually coincide with those studied for treatment response. Studies of other psychiatric disorders such as depression have also reported conflicting results regarding shared genetic bases for etiology and treatment response^56–62^. Furthermore, some of the variants found to be associated with both MDD risk and antidepressant response do it in the same direction (More MDD risk and worse treatment response) while others do it in the opposite (More MDD risk and better response)^60^. Furthermore, PRS composed of risk genetic variants have also yielded inconsistent results as regards predicting SSRI treatment response in MDD patients^26,27,63^.

It is also important to note the role that pharmacokinetic variants apparently play in treatment response^20,21^. These are not specifically considered in PRS methodology since PRS composition is normally derived from GWAS performed in relation to phenotype rather than treatment response. In this regard, it might be interesting to analyze the predictive power of a PRS composed of response-associated rather than risk-associated variants, since these may be not the same. Further analyses of shared and non-shared genetic bases for OCD risk and treatment response will be necessary to enable the design of personalized pharmacological guidelines for OCD in the future.

Our results regarding PRS associations with basal and post Y-BOCS were as expected, since a wide variety of genetic variants have been associated with a higher risk of OCD. Some of these variants are related to general nervous system structure and function (*DISP1, SLC1A1, SLEC6A3, DRD3, 5HT3, BDNF*:^14-17,64^), neurodevelopment (*NGFR* and *CDH9*:^17^), neuroendocrinology (*NR3A1*:^65^), and mitochondrial functioning-related oxidative stress (*MnSOD* and *UCP-2* genes:^66^). Hence, it was expected that a PRS that combines numerous risk variants would predict OCD severity in a sample of patients. Similarly, a PRS composed of risk genetic variants for schizophrenia has emerged as a good predictor of OCD^43^. The associations found between the PRS and both basal and post Y-BOCS seem to suggest that the PRS has the capacity to predict disorder severity independently of pharmacological intervention.

It is important to note that given the predictive capacity of PRS for basal Y-BOCS, including both of them as predictors of post Y-BOCS is statistically redundant. However, including the PRS reduces the model’s statistical strength; thus, *Model 2* (Table 4) presented a worse fit (AIC = 210.324) than the model that did not include the PRS (*Model* 1, Table 4; AIC = 208.677). Hence, despite the collinearity of both the PRS and basal Y-BOCS, since including the PRS makes the model statistically weaker, *Model 1* is a better predictive model than *Model 2*.

Our findings regarding the predictive power of age for treatment response agree with previous results associating a greater age at treatment with a worse response to SSRIs^48^. In addition, the models including age as a predictor (*Model 3*, Table 4; Table S1) showed the best fit (AIC: 205.691; AIC: 203.849). This suggests that age might be an important factor in predicting treatment response in OCD.

A worse response in older OCD patients could indicating a negative correlation between age and neuroplasticity, since it is well known that therapeutic effects of SRI are mediated by neuroplasticity mechanisms and that this decays with age^67,68^. Then, SRI-induced neuroplasticity might not be as optimal in older patients when compared to younger ones. Further research should be developed to clarify this.

As opposed to our results, other studies found response to coadjuvant risperidone in SRI-treated OCD patients to present a positive correlation with age^69^. This might point out to the specificity of our findings for SRI monotherapy. Future research is required to confirm the predictive capacity of age for treatment response, since it could be an important predictor to account for in OCD-treatment.

Analyses of the modulatory role of SLEs at onset showed that these were non-predictors for treatment response when included in the model. However, *t* test analyses revealed differences in the PRS between those reporting and not reporting a SLE at onset. One explanation for this apparent association might be that patients presenting a higher genetic risk for OCD could be more prone to developing the disorder when a SLE occurs. Thus, by itself, a SLE at onset may be insufficient to trigger OCD, but could play a role when other risk factors, such as genetic risk, already exist. Nonetheless, most authors have interpreted SLEs at onset as a reflection of environmentally-explained OCD^12^. Furthermore, compared to SLE-preceded patients, patients not reporting a SLE at onset tend to present a higher frequency of certain clinical characteristics, such as early age at onset or a family history of OCD^70^, which have been attributed to genetic factors^71–73^. These genetic factors might include rare variants with a larger effect than the common variants considered in the PRS, and therefore would not have been captured by our model. Further research is required to clarify the extent to which a SLE at onset can by itself trigger OCD, and to elucidate the real role that SLEs at onset may play in OCD etiology. Furthermore, although pathogenesis and treatment response in OCD are now relatively well established^74,75^, the respective influence of environmental and genetic factors in triggering OCD remains unclear.

It would also be interesting to analyze the modulatory role of other kinds of environmental factors that have consistently been shown to influence the development of OCD, such as lifetime^76^ or childhood^77–79^ trauma. These environmental factors have also been shown to modify the influence exerted by genetic risk on OCD^76^. Our results suggest that patients presenting a more neutral genomic profile could have an environmentally-explained OCD, in which other environmental factors not yet analyzed might be involved in development of the disorder.

Our results concerning SLE associations with age at onset are in agreement with those reported in previous studies, in which non-SLE-preceded patients presented an earlier age of onset^67,80^. In addition, other studies have also found a higher rate of women reporting a SLE at onset^80,81^. This outcome agrees with the literature suggesting that women are more vulnerable to trauma experiences than men^82,83^.

This study presented some limitations. For example, the size of the discovery (*n* = 370 cases; 443 controls) and target sample (*n* = 100) was probably insufficient in terms of statistical strength to enable identification of possible associations^84^. In addition, the target sample presented a somewhat low variability, since it consisted of patients already treated with at least one SSRI. We don’t know if results would have been different in naïve patients without a history of resistance to pharmacological treatment. In the analysis of SLE modulation, the dichotomic nature of our environmental variable might have hindered identification of significant associations. Inclusion of an environmental factor that could be treated as a quantitative variable would enable a richer analysis and facilitate identification of a possible modulatory role.

This is the first study to explore the predictive power of an OCD-risk PRS for treatment response in OCD patients. Our goal was to shed light on the role that polygenic risk may play in treatment response in OCD probands, and the influence of a SLE at onset. Despite the reduced sample size and our negative results on PRS predictive value, some important findings arise from our study. First, the confirmation of age as influencing treatment response should state the importance of early OCD detection and treatment as essential. Secondly, we think that the study of the genetic predictors of SRI response should be expanded to other variants more than those implicated in the risk for affective and anxiety disorders, such as those involved in processes as neuroplasticity or drug-metabolism, while assuming the transdiagnostic aspect of SRI response. Understanding the factors that influence treatment response will enable the development of more individualized treatments that yield a better response and reduce secondary effects and the dropout rate. Further research will be necessary to elucidate the genetic and environmental bases of treatment response in OCD.

## Supplementary information

Supplementary tables

Supplementary Figure (Figure S1)
