# Supplementary material for: Do polygenic risk and stressful life events predict pharmacological treatment response in obsessive compulsive disorder? A gene–environment interaction approach
Source: Transl Psychiatry. 2019 Feb 4;9:70. doi: 10.1038/s41398-019-0410-0 (PMC6362161; doi:10.1038/s41398-019-0410-0)
Supplement: Supplementary file 1 — Supplementary tables [file 41398_2019_410_MOESM1_ESM.docx]

| **Table S1. Basal YBOCS and age as predictors for Post YBOCS.** | | | |
| --- | --- | --- | --- |
|  | *B* | *CI* | *p* |
| (Intercept) | -1.00E-01 | -0.13 to 0.13 | 1.000 |
| Basal Y-BOCS | 0.76 | 0.63 to 0.89 | <.001* |
| Age | 0.17 | 0.05 to 0.30 | .008* |
| Observations | 100 | | |
| R^2^/adj. R^2^ | .575 / .566 | | |
| AIC | 203.849 | | |

The predictive abilities of Basal YBOCS and Age for Post YBOCS are presented as Beta coefficients (β) from the regression as well as with the proportion of variance explained by the model (R^2^/Adj. R^2^). The model fit is also reported by the Akaike Information Criterion (AIC).

Y-BOCS, Yale-Brown Obsessive Compulsive Scale; PRS, Polygenic Risk Score.

*p<.05

| **Table S2. Differences in PRS and age at onset between SLE-preceded and non SLE-preceded patients** | | | | | | | | | | | | | | | | | | | |  |  |
| --- | --- | --- | --- | --- | --- | --- | --- | --- | --- | --- | --- | --- | --- | --- | --- | --- | --- | --- | --- | --- | --- |
|  | *SLE-preceded* | | | | |  | | | *Non-SLE preceded* | | | | |  | *t* | | *df* | *P* | |  |  |
|  | Mean | | S.D | | | | | | Mean | S.D. | | | |  |  |  | | | | | |
| PRS | .000272 | .000581 | | | | | -.000063 | | | | .000536 | | |  | 2.96 | | 98 | .004* |  |  |  |
| Age at onset | 24.38 |  | | 7.38 |  | |  | 19.78 | | | | 11.14 |  | | 2.29 | | 98 | .024* | | |  |

Results from t-test analysis performed for PRS and Age at onset in relation to SLE are displayed. The table shows mean and standard deviation (S.D.) values for PRS and Age at onset in patients reporting a SLE at onset of the disorder and patients not reporting it. T values, degrees of freedom (df) and p-values can be seen for both t-tests performed.

PRS, Polygenic risk score; SLE, Stressful life events.

**p*<.05

| **Table S3. Differences in sex between SLE-preceded and non-SLE preceded patients.** | | | | | | | | |
| --- | --- | --- | --- | --- | --- | --- | --- | --- |
|  | SLE-preceded |  | | non-SLE preceded |  | *Χ^2^* | *df* | *P* |
|  |  | |  |  |  | 4.93 | 1 | .039* |
| Men n (%) | 17 (42.5) | |  | 39 (65) |  |  |  |  |
|  |  | |  |  |  |  |  |  |
| Women n (%) | 23 (57.5) | |  | 21 (35) |  |  |  |  |
|  |  | |  |  |  |  |  |  |
| Total n (%) | 40 (40) | |  | 60 (60) |  |  |  |  |

Results from chi square tests performed for sex and SLE are reported. The table shows the number and proportion of men and women reporting a SLE at the onset of the disorder and not reporting it. Chi square value (*Χ^2^*), degrees of freedom (df) and p-values are displayed.

SLE, Stressful life events.

*p<.05
