# Supplementary material for: Do polygenic risk and stressful life events predict pharmacological treatment response in obsessive compulsive disorder? A gene–environment interaction approach
Source: Transl Psychiatry. 2019 Feb 4;9:70. doi: 10.1038/s41398-019-0410-0 (PMC6362161; doi:10.1038/s41398-019-0410-0)
Supplement: Supplementary file 2 — Supplementary Figure (Figure S1) [file 41398_2019_410_MOESM2_ESM.pdf]

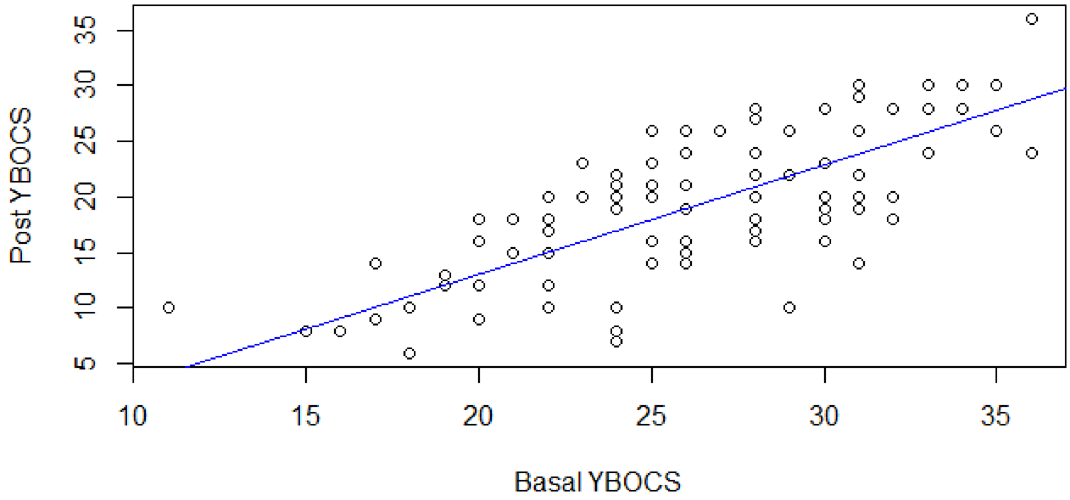


**Figure S1**. Scatterplot of Post YBOCS scores for different Basal Y-BOCS scores. Trend line (blue) indicates regression model fit.
